# Intrathecal Drug Delivery (ITDD) systems for cancer pain

**DOI:** 10.12688/f1000research.2-96.v4

**Published:** 2014-07-28

**Authors:** Gaurav Bhatia, Mary E Lau, Katharine M Koury, Padma Gulur

**Affiliations:** 1 Department of Anesthesia, Critical Care, and Pain Medicine, Massachusetts General Hospital, Boston MA, 02114, USA

## Abstract

Intrathecal drug delivery is an effective pain management option for patients with chronic and cancer pain. The delivery of drugs into the intrathecal space provides superior analgesia with smaller doses of analgesics to minimize side effects while significantly improving quality of life. This article aims to provide a general overview of the use of intrathecal drug delivery to manage pain, dosing recommendations, potential risks and complications, and growing trends in the field.

## Introduction

Intrathecal drug delivery (ITDD or IDD) is a method which provides analgesia by a direct continuous infusion of medication into the cerebrospinal fluid (CSF). The rationale is based on the concept that direct delivery into the CSF within the intrathecal space avoids crossing the blood-brain barrier and allows delivery of smaller doses than oral, intravenous, transdermal or epidural drug administration ^1^. The spinal cord is a key anatomical site for pain processing and more specifically the dorsal horn of the spinal cord within the intrathecal space. There are various receptor targets within the spinal column that can be utilized in relieving pain. These receptors include opioid (mu, kappa, and delta), GABA, alpha-2, dopaminergic, NMDA as well as sodium and calcium channels ^2^. The advantage of delivering the medication directly into the CSF in smaller dosages to target the specific receptors is that it may reduce the systemic side effects and the direct delivery to the site of action allows a more rapid and effective response. As a popular alternative for patients experiencing cancer or chronic pain, intrathecal analgesia has become a therapeutic option for patients who have exhausted all other treatment avenues as well as patients experiencing side effects from their current treatment options. The system uses a small pump that is surgically placed under the skin of the abdomen and delivers medication through a tunneled catheter directly into the intrathecal space ^3^. The pump is refilled periodically through a subcutaneous access port in an office setting utilizing a sterile technique. The pump can be programmed to be a fixed-rate, constant flow device or a variable rate pump with the option of boluses via patient controlled analgesia programming using a wireless radiofrequency transmitter given to the patient ^1^. In contrast to more conservative oral or intravenous delivery methods, ITDD is becoming increasingly popular for its efficacy in managing cancer and chronic pain. This review will specifically target ITDD uses for patients with cancer-related pain although its use has also been very popular in patients with chronic non-cancer pain.

## Cancer pain: epidemiology

The incidence of deaths due to cancer has been estimated to be 6.6 million people globally each year ^4^. Pain is often considered as the most feared symptom amongst individuals living with cancer and it can occur at any point during the course of the illness. Pain is often also the first sign of malignancy ^4^. Pain is also often associated with the treatment of cancer and it can present itself as both acute and chronic pain. Furthermore, advancement of the disease process can lead to pain or make the existing pain worse. It is difficult to estimate the prevalence of cancer pain because of a lack of standardization in definitions of pain and in the measures used to assess it ^5^. The other factor that makes it challenging in estimating the prevalence is the heterogeneity of nociceptive and neuropathic pain conditions ^6^. About 50% of patients will report pain as a symptom at the time of cancer diagnosis and early in the course of disease ^6^. Its prevalence increases to 75% or more at advanced stages. A strategy should be devised to evaluate the prevalence of pain in patients with cancer by separating the pain related to the disease process, to its treatment, or to unrelated causes.

## Prevalence of pain related to cancer

According to a systematic review of the past 40 years by van den Beuken *et al.* published in the Annals of Oncology in 2007 ^4^, the range of reported prevalence of pain is highest for the following tumors:

- Head and neck (67–91%)
- Prostate (56–94%)
- Uterine (30–90%)
- Genitourinary (58–90%)
- Breast (40–89%)
- Pancreatic (72–85%)

## Treatment options

The various treatment options for cancer pain generally depend on the location, character, duration, its origin, and the success or failure of previous and ongoing treatments. Treatment options for cancer-related pain include oral non-steroidal anti-inflammatory drugs, oral opioid analgesics, neuropathic pain medications, parenteral opioids, peripheral nerve blocks, neurolysis such as celiac plexus blocks, local anesthetic injections, spinal cord stimulation, continuous epidural analgesia, and intrathecal pumps. In most cases, multiple forms of treatment are used for the most effective treatment combination. Opiates such as morphine, dilaudid, fentanyl, and sufentanil are most commonly administered via the intrathecal drug delivery system for cancer-related and non-malignant pain ^7^. There is increasing evidence that intrathecal opioids are superior to oral delivery in malignant pain, especially when narcotic dosage is limited by its side effects ^1^. It is believed that this advantage results from delivering extremely small doses on target receptors in the spinal column ^8^.

There are other agents also available to treat neuropathic pain, spasticity related pain, sympathetic pain, and visceral pain (see Table 2 for examples). In addition, ITDD systems can also be used to deliver chemotherapy agents such as floxuridine and methorexate for the treatment of primary or metastatic cancer. Table 2 below lists the most commonly targeted receptors and the medications used for each receptor.

**Table 1.** Conversion ratios of morphine. All other drugs are converted to Morphine Equivalent Dose (MED) to allow equianalgesic conversions.

Route of administration	Conversion ratio
Oral	300
Intravenous	100
Epidural	10
Intrathecal	1

**Table 2.** Receptor targets for pain medications ^2^.

Receptors targeted	Intrathecal drug delivery system medication(s)
Opioid (mu, delta, kappa)	Morphine, hydromorphone, fentanyl, sufentanil, methadone
GABA	Baclofen, midazolam
Sodium channel receptors	Local anesthetics such as bupivicaine, levobupivacaine, ropivacaine
NMDA	Ketamine, methadone
Calcium channel receptors	Ziconotide
Alpha-2 receptors	Clonidine, dexmedetomidine
Other agents (rarely used)	Adenosine, gabapentin, ketorolac, neostigmine, octreotide

**Note:** The only medications that are FDA approved for ITDD are: Morphine Sulfate, Baclofen, Ziconotide, Floxuridine, and Methotrexate ^9^.

One of the most important clinical decisions a provider has to make is what agent to use in the intrathecal pump and in some cases multiple forms of treatment are used for the most effective treatment combination. In this review, we looked at the recommendations of The Polyanalgesic Consensus Conference (PACC) 2012 which lists the algorithms for intrathecal therapies in both neuropathic and nociceptive pain ( Table 3 and Table 4). The algorithm lists the medications that are arranged in a hierarchy on the basis of evidence of efficacy and safety. The approaches are listed from Line 1 (first line approach) to Line 5 (a more advanced approach if previous approaches are unsuccessful).

**Table 3.** 2012 polyanalgesic algorithm for intrathecal (IT) therapies in neuropathic pain ^7^.

**Line 1**	Morphine	Ziconotide		Morphine + bupivicaine
**Line 2**	Hydromorphone	Hydromorphone + bupivicaine OR Hydromorphone + clonidine		Morphine + clonidine
**Line 3**	Clonidine	Ziconotide + opioid	Fentanyl	Fentanyl + bupivicaine OR fentanyl + clonidine
**Line 4**	Opioid + clonidine + bupivicaine		Bupivicaine + clonidine	
**Line 5**	Baclofen			

**Note:** Line 6-Experimental agents: Gabapentin, Octreotide, Conpeptide, Neostigmine, Adenosine, XEN 2174, AM 336, XEN, ZGX 160 (ongoing experiments in animal models).

**Table 4.** 2012 polyanalgesic algorithm for intrathecal (IT) therapies in nociceptive pain ^7^.

**Line 1**	Morphine	Hydromorphone	Ziconotide	Fentanyl
**Line 2**	Morphine + bupivicaine	Ziconotide + opioid	Hydromorphone + bupivicaine	Fentanyl + bupivicaine
**Line 3**	Opioid + clonidine			Sufentanil
**Line 4**	Opioid + clonidine + bupivicaine		Sufentanil + bupivicaine or clonidine	
**Line 5**	Sufentanil + bupivicaine + clonidine			

**Note:** Line 6-Experimental agents: Gabapentin, Octreotide, Conpeptide, Neostigmine, Adenosine, XEN 2174, AM 336, XEN, ZGX 160 (ongoing experiments in animal models).

### Non-malignant pain and other uses

A retrospective study ^10^ involving 120 patients with non-malignant pain syndromes found that the patients benefited from the intrathecal opiate therapy and the average pain reduction was 67.4% after 6 months from implantation. Over ninety percent of the patients were satisfied with the therapy and 81% reported an improvement in their quality of life.

Besides opiates, other medications such as baclofen (GABA agonist) for spasticity related pain, ziconotide (calcium channel blocker) for severe chronic neuropathic pain, bupivicaine (amide local anesthetic), clonidine (alpha-2 agonist), and ketamine (NMDA antagonist) have also been used in various instances and in specific patient groups. In addition, ITDD systems can also be used to deliver chemotherapy agents such as floxuridine and methorexate for the treatment of primary or metastatic cancer ^9^.

One of the most common uses of the ITDD system is the delivery of baclofen for spasticity related pain ^3^. Baclofen is a GABA-A receptor agonist which can have a prominent effect on the motor tone via direct hyperpolarization of the motor horn cells. It is most often utilized in patients suffering from spasticity, cerebral palsy, amyotrophic lateral sclerosis, Stiff-Pearson syndrome, and those suffering from brain and spinal cord injuries ^7^.

## Intrathecal drug delivery systems

**Figure 1.**
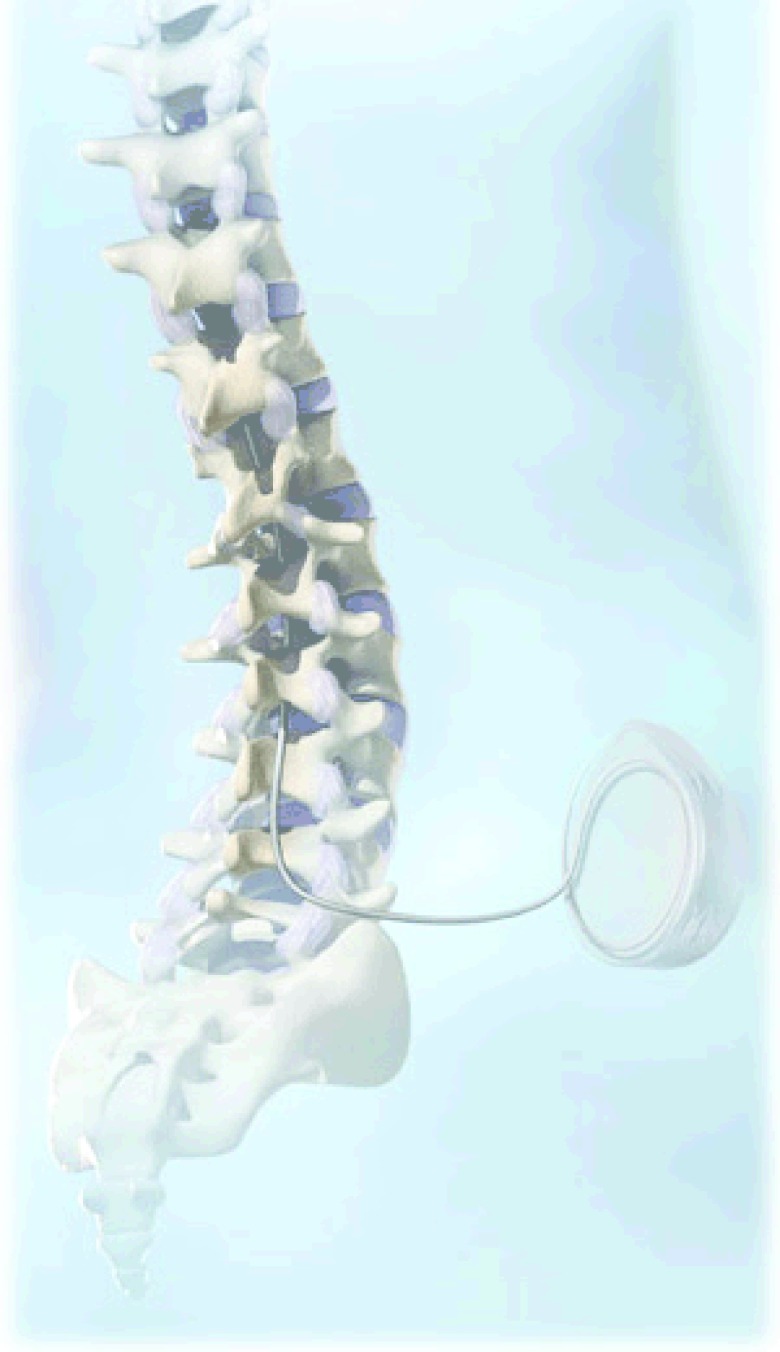
Image of the spine illustrating placement of the catheter into the intrathecal space which is connected to an implanted pump containing the drug reservoir. Reprinted with the permission of Medtronic, Inc.© 2010.

One option, intrathecal analgesia, has become a therapeutic option for patients who have exhausted all other treatment avenues as well as patients experiencing side effects from their current treatment options ^3^. In intrathecal drug delivery or ITDD, medication is delivered directly into the CSF in the intrathecal space ( Figure 1); therefore, a substantially smaller dose of medication is required as compared to the oral or intravenous route. With lesser dosage of the medication and a more direct route to the pain processing center in the spinal cord, the patient generally experiences superior analgesia with fewer side effects such as nausea, pruritis, erythema, flushing, constipation, etc. However, intrathecal delivery of medication also poses significant risks such as respiratory depression, infection, bleeding, epidural/spinal hematomas, spinal cord injury during initial catheter placement, wound dehiscence, pocket seromas, pump malfunction, and costs.

There have been various publications and consensus guidelines in regards to a screening trial before proceeding with pump implantation ^2, 3, 7^. Previously, trialing was considered to be the standard of care and critical in evaluation of a patient’s response and potential side effects from a certain agent. However, more recently, the PACC 2012 expert panel has called it debatable specifically in cancer-pain patients ^7^. The issues of opioid-induced hyperalgesia (OIH) or disease progression is difficult to assess from a single-shot trial or even with a brief 72–96 hour infusion. A trial may also lead to underestimation of the failure rate and potential side effects. Still, the rationale for performing trials is based on the intuitive basis that it best mimics the system that would eventually be implanted ^7^. Generally speaking, there is currently no solid base to either refute or adopt preimplantation trials but it is generally recommended although not required ^2^. The British Pain Society expert panel suggested that trials should always be performed before the implantation of an intrathecal drug delivery system ^7^.

There is a spectrum of intrathecal system options that range from a percutaneous catheter/external pump to a totally implanted system. Choice is dependent on a number of factors including, but not limited to, life expectancy, cost and availability of professional expertise and patient’s wishes and comfort level.

### Percutaneous

In general, percutaneous refers to a medical procedure in which a catheter or a medical device is introduced into the body via needle-puncture of the skin. This approach is favored because the catheter is easy to place (fluoroscopy can be used to confirm the position of the catheter and to maneuver it into the desired location) and is suitable for patients with limited life expectancy. The catheter is then attached to an external pump which delivers the medication directly into the intrathecal space. However, it is generally considered only a temporary option due to limitations such as frequent monitoring for infection, catheter migration, and patient’s immobility. The risks from this procedure are similar to performing a spinal anesthetic which includes bleeding, infection, headache, spinal cord damage, etc.

### Fully implanted

Fully implanted drug delivery systems are suitable for long-term use. Mobility and functional activity are not particularly adversely affected by these systems. The implantation is performed by a skilled health care provider in an operating room under monitored anesthesia care with local anesthetic infiltration or general anesthesia. The patients require specialized care with a full multi professional infrastructure including regular follow-up appointments for pump refills. The pump can be programmed to be a fixed-rate, constant flow device or a variable rate pump with the option of boluses via patient controlled analgesia programming using a wireless radiofrequency transmitter given to the patient. Fixed rate delivery systems are less expensive than variable rate delivery systems but lack flexibility of medication delivery based on the need of a patient. These systems have a larger reservoir volume which allows for longer intervals between refills. In cases of suspected or actual medication overdose or implant malfunction, the pump’s drug reservoir has to be emptied and the device is interrogated by a designated health care professional.

## ITDD devices

There are two systems available for implantable intrathecal devices–those that are programmable and those that are fixed-rate. In addition, some systems come with a bolus option that gives patients more control in managing their treatment. The needs of the patient as well as the suitability of the system for use with selected drugs need to be considered when deciding between the options.

### Programmable devices

**Figure 2.**
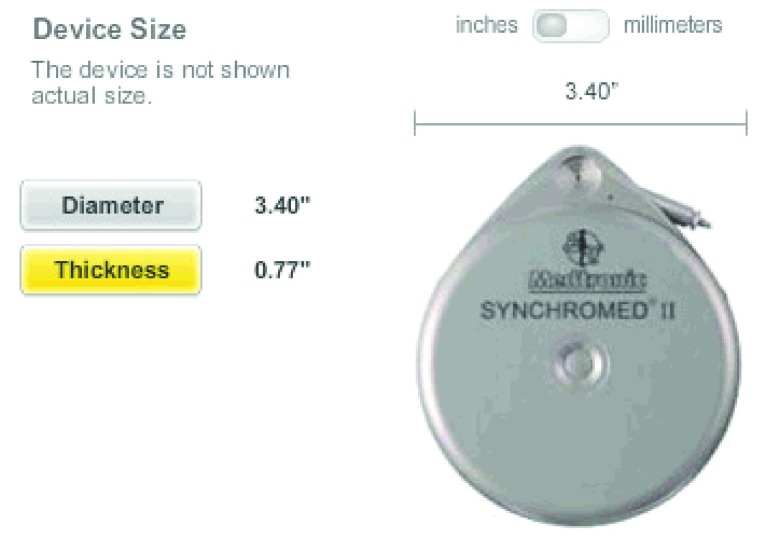
Image of the SynchroMed II Pump with specifications. Pump is programmable to deliver a specific amount of medication at different times and can be increased or decreased depending on the individuals needs. Reprinted with the permission of Medtronic, Inc.© 2009.

**Figure 3.**
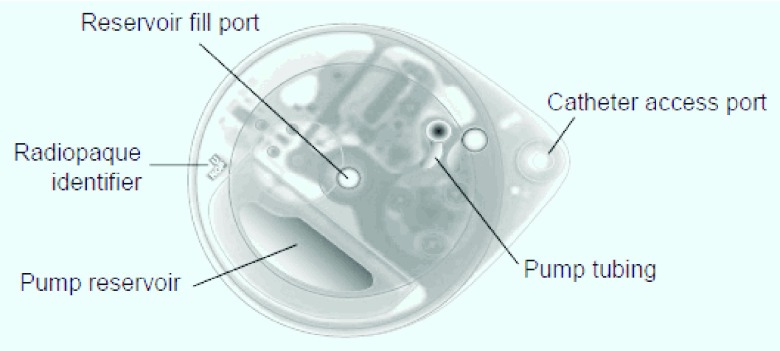
X-ray of the SynchroMed II Pump. The pump has 20 and 40 cc reservoirs to extend the time between refills. Drug is refilled via the reservoir fill port. Reprinted with the permission of Medtronic, Inc.© 2010.

Programmable devices such as Medtronic’s SynchroMed system deliver a specific amount of medication intermittently ( Figure 2 and Figure 3). These systems are preferred by providers because they allow drug dosages to be changed without invasive intervention as the disease progresses and/or the patient builds tolerance to the medication. In cases of suspected or actual medication overdose or implant malfunction, the pump can be interrogated and deactivated without having to empty the drug reservoir. However, since it is battery driven, the life of this pump is usually 5–8 years and a surgical revision is required to implant a new pump. In addition, regular attendance for refilling is required because the drug reservoir tends to be smaller in volume than those found in fixed-rate devices.

### Fixed-rate devices

Constant flow systems such as Codman’s 3000 delivers a continuous amount of medication into the intrathecal space. These devices are favorable because they are generally less expensive, do not require a battery to operate and possess a larger drug reservoir that allows the drug to be delivered for a longer period of time before requiring a refill. However, constant rate devices are disadvantageous because they don’t give the flexibility to change the programming or a patient controlled bolus option for additional analgesia.

### Devices with bolus option

The Medtronic SynchroMed II infusion system comes with a Patient Therapy Manager (PTM) which is a trademark of the Medtronic Corporation ^11^. It is a remote hand-held device which communicates with the implanted pump and allows the patient to self administer boluses based on the medication limits which are pre-programmed by the clinical provider. This system gives patients the ability to activate a bolus option when adequate pain relief is not achieved. The clinical care provider has the ability to set the number of boluses a patient may receive in a 24 hour period. There is a lockout system in place with the programmer that does not allow the patient to self activate the bolus option more than what it is allowed based on the settings created by the clinical provider. This allows for flexibility for the patients who have more intimate needs to control their pain based on certain body positions, movement, time of the day, and/or progression of their underlying disease. There are several safety options built in to the device such as the maximum number of boluses the patient may self administer in a 24 hour period, maximum dosage of the total drug in a 24 hour period, lockout in-between bolus activation, and the duration of time during which a bolus is administered.

## Placement ^12^

The placement of ITDD consists of two parts: 1) placement of the catheter in the intrathecal space surrounding the spinal cord and 2) placement of the pump/reservoir in the abdomen/flank region. The pump is implanted in the lower abdomen and is attached to the intrathecal catheter which is tunneled under the skin from the patient’s back towards the abdomen. The procedure requires two surgical incisions: 1) Lower back where the catheter is inserted into the intrathecal space and is secured to the fascia. 2) The lower abdominal wall to create a pocket for the pump. The procedure generally takes 3 to 4 hours from start to finish.

### Patient selection

Patient selection is an important consideration. Patients who are good candidates for ITDD therapy include those that have significant side effects from oral or IV opioids which hinders titration of these agents to adequate analgesia. Another criterion includes patients who do not achieve adequate analgesia in spite of significant doses of oral opioids. These pumps are implanted by pain physicians and neurosurgeons. When implanted by pain physicians, they continue to manage the pump as well. Neurosurgeons primarily implant the pump which they then send back to pain specialists or palliative specialists to manage. It has been shown that ITDD for pain management can actually help patients on cancer therapies such as chemotherapy or radiation ^13^. This is because ITDD has less adverse effects than other pain treatments, so patients can endure more aggressive levels of chemotherapy or radiation.

### Preoperative preparation

Prior to the procedure, a thorough evaluation is performed by the anesthesiologist to ensure patient’s ability to undergo surgery and anesthesia. Physical exam, blood tests, electrocardiogram, and chest x-rays may be requested by the provider. Current systemic use of analgesics is discontinued as well as anticoagulants and antiplatelet therapies. The position of the pump is proposed and agreed upon by the patient and provider keeping patients’ comfort, activity, need for future procedures and rehabilitation matters in consideration. The choice of medication is dependent on the success of past and present medication choices and acceptable side effects. This is usually pre-determined from either previous efficacy of a particular medication or during a trial period.

### Operating room procedure

Implantation of an ITDD system is a minor surgical procedure that is carried out in the operating room using aseptic precautions, including skin preparation, sterile draping, and the use of full surgical attire. The patient is positioned in a lateral decubitus position with the patient’s side for the pump pocket nondependent ^12^. Real time fluoroscopy is used to identify the ideal location for placement of the catheter via a needle provided by the device manufacturer. A small skin incision is made in the middle of the back to expose the fascia over the bony arch (lamina) of the vertebra and the catheter is placed in the subarachnoid, or intrathecal space and then anchored into the fascia to prevent migration.

Once the catheter is in place and secure, a tunneling device is used to pass the catheter under the skin from the spine to the abdomen where the pump will be implanted. A 4–6 inch skin incision is made in the side of the abdomen below the waistline. The surgeon then creates a pocket for the pump between the skin and muscle layers and the catheter is attached to the pump. Once the connector is attached to the pump, the pump is correctly positioned under the skin and secured to the thick fascia layer overlying the stomach muscles using suture loops. A space inside the pump called the reservoir is filled with the desired medication and the pump is programmed. The incisions in the back and abdomen are closed with sutures or staples and a dressing is applied. The patient is taken to the recovery area and is monitored per the standard requirements of the institution before being discharged.

### Post-operative

Often antibiotics are prescribed to prevent complications and a one week follow-up is recommended. There may be temporary restriction on daily activities to allow for healing of the wound and pump to settle. Once healed, no special care is necessary.

## Dosing and titration

As mentioned previously, ITDD delivers medicine directly into the cerebrospinal fluid, bypassing the route that oral medication takes through the body. Generally 1/100 ^th^–300 ^th^ of the amount of medication is used with the pump delivery as compared with the amount when taken orally. A survey conducted among a small group of physicians highly-experienced in ITDD revealed that morphine was the more frequently used and prescribed choice of medication, both alone and in combination with other drugs ^14^. The concentrations and daily dosages administered of medications varied from patient to patient. Providers often adjusted the dosages of drugs or drug combinations by a fixed percentage, between 10% and 20%, whether increasing or decreasing the dosage. An average of five to eight adjustments in dosage was completed before a different drug or drug combination was introduced in cases of inadequate analgesia ^13^.

## Efficacy

Studies have suggested that ITDD is effective in providing pain relief to 60–80% of patients experiencing chronic malignant pain ^7^. Long-term intrathecal application of opioid medications in cases of cancer pain is substantially more effective than its systemic application. Intrathecally applied opioids exert a strong analgesic effect via spinal receptors, without significantly influencing motor, sensory, and sympathetic reflexes. It has been shown to improve patients’ overall mood and reduces the incidence of systemic side effects. As a result, patients have reported an improvement in quality of life and the ability to participate more fully in daily activities. Furthermore, a randomized controlled study illuminates the efficacy of ITDD over comprehensive medical management in treating refractory cancer pain ^13^. Patients receiving ITDD not only had better pain control and relief, but also had improved survival, a reduction in drug toxicity, and fewer drug side effects ^13^.

## Complications

As with any surgical procedure, serious complications may arise post-implant that require immediate attention and perhaps the removal of the device.

### Drug related complications

A retrospective study ^15^ showed that the most common complication was patients’ adverse reaction to a drug. Pharmacological complications were the most common immediately post-implant and generally subsided as treatment continued. Serious complications include respiratory depression/arrest, anaphylaxis, and meningitis with introduction of contaminated solution. It was also found that certain opioids (the drugs of choice) were more prone to complications than others. For example, the incidence of tip catheter granulomas is higher with morphine and hydromorphone as compared to fentanyl which has been upgraded to a first-line drug in the algorithm due to that specific advantage ^7^. Table 5 below lists the most common drug related adverse reactions and it is compiled based on the data presented in the PACC 2012.

**Table 5.** Drug related adverse reactions to medications delivered via ITDD ^2^.

Adverse reaction	Associated medications
Peripheral edema	Opioids
Hormonal changes	Opioids
Respiratory depression/somnolence	Opioids, benzodiazepines, local anesthetics, baclofen
Granuloma	Opioids (except fentanyl)
Hyperalgesia/tolerance/withdrawal	Opioids, baclofen
Immune suppression	Opioids
Psychosis, suicidality, hallucinations, confusion	Ziconotide, clonidine, baclofen
Urinary retention, weakness, hypotension	Opioids, local anesthetics
Demyelination, necrotizing lesions	Ketamine, dexmedetomidine

### Device related complications

Catheter and pump related malfunctions are one of the main sources of complications. In fact, one study ^15^ found catheter related complications as the most common cause of repeat surgery. Despite suturing the catheter to the underlying fascia, coiling of the tube and leakage at the connection site remains a problem. A study reviewing complications from long-term intrathecal drug therapy found that the annual rate for complications requiring surgical procedure was 10.5%, with 35% being pump related and 65% catheter related ^2^. Errors in pump programming have also been reported and resulted in incorrect flow rates. Device related complications have led to infections (meningitis), pockets abscesses, bleeding, pain and discomfort, and blood or fluid in the pump’s pocket. Table 6 lists the most common device related side effects.

**Table 6.** Device related side effects ^2^.

Device related side effects:
Infection/meningitis
Post-dural puncture headaches/CSF leak
Catheter tip granuloma formation
IT catheter and pump malfunctions
Pocket site seroma formation, bleeding, pain and discomfort

### Patient related complications

Although rare, patient induced complications generally involve infections at implant location. Immune cells form a mass, known as a granuloma, around the catheter tip to wall off foreign substances. However, the incidence of infections in all cases reviewed during a study was 0.7% per year ^16^, with all infections appearing within the first three months post-implant. Factors that increased the potential risk of complication included psychological problems, obstructive sleep apnea, immunosuppression, smoking, diabetes active infections, bleeding disorders, and concurrent anticoagulation therapy ^2, 7^. A data analysis found that one successful measure to further decrease the chance of infection was adherence to guidelines and recommendations for surgical site infections ^17^.

### Compounding of medications and its considerations

The FDA and the United States Pharmacopoeia have issued standards on compounded sterile products that have clinical, legal, and practical significance ^7, 9^. These standards apply to compounding of solutions by various routes, including intrathecal administration. There are strict provisions in place for considerations such as: training of personnel, a segregated sterile compounding area, air quality of the compounding area, certification and calibration of equipment, a cleaning and disinfection program, and a quality assurance program ^7^. Any violation of any of the provisions can result in a civil and criminal law suit in addition to the suspension of the privileges to dispense compounded medications. An outbreak of fungal meningitis resulted in 2012 when several lots of contaminated methylprednisolone vials were used for epidural steroid injections in various states which were obtained from a compounding pharmacy. Shortly following the outbreak, the device manufacturer Medtronic released an "Urgent Medical Device Safety Notification" in November 2012 discouraging the use of unapproved drugs with its delivery system ^9^.

## Cost effectiveness

With estimates of pain care treatment costs exceeding $1 billion annually in the United States, cost and efficacy are important factors in clinical decision making. Although the initial cost of intrathecal drug delivery is substantially more, maintenance costs over time are significantly lower than conventional routes of administration. Cost analyses by the group concluded that intrathecal delivery is the most cost-effective route of opioid administration for patients who require long-term management of cancer (≥ 3–6 months) or nonmalignant pain (≥ 11–22 months) ^18^.

An analysis by Kumar *et al.* showed that intrathecal therapy is a cost-effective method of treating chronic nonmalignant pain caused by failed back syndrome and that the break-even point occurs at 28 months, after which conventional pain therapy is the more expensive treatment option ^19^. A separate analysis by Scott Guillemette from Ingenix Consulting on patients suffering from failed back surgery syndrome concluded with similar results. Despite the higher upfront costs, patients utilizing intrathecal drug delivery returned to ‘normal living’ more quickly than those with conventional therapy and the break-even point between the two points occurred between months 19 and 20 ^20^.

## Conclusion

The treatment of pain poses an ongoing challenge to the healthcare field and better prevention, assessment and treatment of pain is needed. Intrathecal drug delivery can become a critical component of this transformation in treatment when more conservative forms of pain management have failed to provide adequate pain relief. In comparison to more conservative delivery methods such as drugs administered orally or intravenously, ITDD is more effective because the medication is directly introduced into the subarachnoid space ^8^. The spinal cord is a key anatomical site for pain processing and more specifically the dorsal horn of the spinal cord within the intrathecal space ^5, 8^. The advantage of delivering the medication directly into the CSF in smaller dosages is that it may reduce the systemic side effects and the direct delivery to the site of action allows a more rapid and effective response. As a popular alternative for patients experiencing cancer or chronic pain, intrathecal analgesia has become a therapeutic option for patients who have exhausted all other treatment avenues as well as patients experiencing side effects from their current treatment options. However, there are limitations such as adequate patient selection, ability to tolerate the procedure, and costs. There are also potential complications related to the device, drug, and procedure that may require hospitalization and immediate removal/revision of the device. Despite numerous studies involving existing and novel drugs, only limited data is available to address the issue of medication safety, efficacy, stability and compatibility with the intrathecal drug delivery system. As this option becomes more popular with patients and physicians, future research should focus on cross-site studies that would provide a more accurate outlook on this type of delivery.
