# Supplementary material for: Intrathecal Drug Delivery (ITDD) systems for cancer pain
Source: F1000Res. 2014 Jul 28;2:96. Originally published 2013 Mar 28. [Version 4] doi: 10.12688/f1000research.2-96.v4 (PMC3892916; doi:10.12688/f1000research.2-96.v4)
Supplement: Peer review history

# Peer review: Referee response for version 3

**DOI:** 10.5256/f1000research.1897.r1577

**Published:** 2013-08-27

**Authors:** Marcin Chwistek

**Affiliations:** 1 Department of Medical Oncology, Fox Chase Cancer Center, Philadelphia, PA, USA

I have read this submission. I believe that I have an appropriate level of expertise to confirm that it is of an acceptable scientific standard.

# Peer review: Referee response for version 3

**DOI:** 10.5256/f1000research.1897.r1428

**Published:** 2013-08-09

**Authors:** Michael Erdek

**Affiliations:** 1 Department of Anesthesiology & Critical Care Med, John Hopkins University, Baltimore, MD, USA

I have read this submission. I believe that I have an appropriate level of expertise to confirm that it is of an acceptable scientific standard.

# Peer review: Referee response for version 2

**DOI:** 10.5256/f1000research.1520.r1006

**Published:** 2013-06-17

**Authors:** Marcin Chwistek

**Affiliations:** 1 Department of Medical Oncology, Fox Chase Cancer Center, Philadelphia, PA, USA

This is an informative review (written with the general practitioner in mind) that provides the basic information and rationale for using ITDD for the treatment of cancer pain.  

I have one major reservation and a few suggestions: 

1. Cancer pain can be managed by a variety of practitioners; in the majority of situations it will fall under the purview of the patient’s primary oncologist or a primary care physician, who typically are unfamiliar with ITDDs. It would be very helpful, in my view, to add a paragraph that would outline in more detail when one should start thinking about referring a patient for consideration of ITDD? What criteria does the patient need to meet? E.g. life expectancy? Whom such patients should be referred to? Who will manage the pump after the implantation? How is it going to affect further treatment such as chemotherapy or radiation? What kind of treatment break one can expect? This kind of information is crucial for the treating oncologist. Some of this information is already in the text, however it would be very useful to organize it better and add some more specific and practical information. Perhaps in a form of a table?   

A few clarifications may be needed:

Table 2: 

- Is Meperidine still used as an opioid in ITDD? Its clinical use, especially in cancer pain, has been basically eliminated. 

- NMDA receptor – consider adding Methadone. Methadone is an opioid but also has NMDA activity

Page 7: 

- In the US, the commonly used term is  “cancer pain”, instead of “carcinoma pain”

Marcin Chwistek, MD FAAHPM

I have read this submission. I believe that I have an appropriate level of expertise to confirm that it is of an acceptable scientific standard, however I have significant reservations, as outlined above.

# Peer review: Referee response for version 2

**DOI:** 10.5256/f1000research.1520.r1004

**Published:** 2013-06-14

**Authors:** Michael Erdek

**Affiliations:** 1 Department of Anesthesiology & Critical Care Med, John Hopkins University, Baltimore, MD, USA

I don't see that the question of the difference in thickness between the 20cc and 40cc pumps was addressed.

I have read this submission. I believe that I have an appropriate level of expertise to confirm that it is of an acceptable scientific standard.

# Peer review: Referee response for version 1

**DOI:** 10.5256/f1000research.1238.r905

**Published:** 2013-04-22

**Authors:** Arun Bhaskar

**Affiliations:** 1 Research Division, The Christie NHS Foundation Trust, Manchester, UK

This article gives a broad overview of the various types of ITDD systems used in the management of complex cancer pain. It also discusses the advantages, the common side-effects and complications, the technique and cost-effectiveness of using ITDD systems. Overall the article is useful in providing an understanding for doctors and nurses not familiar with the intrathecal drug delivery systems.  

The article is written at a basic level and although it is useful to beginners and people who are not well versed in ITDD, it does not deliver much to experienced practitioners. I have reservations as I feel that this review article would have been of a higher standard if it was more comprehensive.

I have read this submission. I believe that I have an appropriate level of expertise to confirm that it is of an acceptable scientific standard, however I have significant reservations, as outlined above.

# Response

**Published:** 2013-06-10

**Authors:** Padma Gulur

**Affiliations:** Anesthesiology, Pain Medicine, UC Irvine Medical Center, USA

Thank you very much for your response. This article was written for general practitioners to gain an understanding of ITDD systems for cancer pain.

# Peer review: Referee response for version 1

**DOI:** 10.5256/f1000research.1238.r885

**Published:** 2013-04-10

**Authors:** Michael Erdek

**Affiliations:** 1 Department of Anesthesiology & Critical Care Med, John Hopkins University, Baltimore, MD, USA

Page 3: There can be a need for increasing doses of opioids as tolerance develops.

Page 5: There is a difference in thickness of the 20cc and 40cc pumps.

Page 6: The lamina is not routinely exposed unless laminotomy leads are being placed.

Page 7: Reference Smith TJ *et al. *(2002) *J Clin Oncol.* for efficacy.

Page 7: Reference Follett K *et al*. (2004) *Anesthesiology* for complications.

Page 7: (Table 5) – Ziconotide is not an opioid.

I have read this submission. I believe that I have an appropriate level of expertise to confirm that it is of an acceptable scientific standard, however I have significant reservations, as outlined above.

# Response

**Published:** 2013-06-10

**Authors:** Padma Gulur

**Affiliations:** Anesthesiology, Pain Medicine, UC Irvine Medical Center, USA

Thank you very much for your response. 

**Page 3: There can be a need for increasing doses of opioids as tolerance develops.** We understand and agree with your comment; however, we are unsure as to where you feel this statement should to be added within the article. 

**Page 5: There is a difference in thickness of the 20cc and 40cc pumps.** We understand and agree with your comment; however, we are unsure as to where you feel this statement fits in the article.

**Page 6: The lamina is not routinely exposed unless laminotomy leads are being placed.** **6.2 Operating room procedure** **- **We have changed the sentence to say that the incision is made to expose the fascia over the bony arch (lamina).

**Page 7: Reference** **Smith TJ *et al. *(2002) *J Clin Oncol.* for efficacy** **.**  A discussion of this article has been added into the efficacy section to show that ITDD is a better system of pain management than comprehensive medical management for patients with cancer pain.****

**Page 7: Reference Follett K *et al*. (2004) *Anesthesiology* for complications.**  After reviewing this article, it has been added into our article that in order to further decrease the chances of infection, there must be an adherence to guidelines and recommendations regarding surgical site infections. 

**Page 7: (Table 5) – Ziconotide is not an opioid. **             Ziconotide has been taken out of the chart.
